# A model of hemodialysis after acute kidney injury in rats

**DOI:** 10.1186/s40635-023-00583-7

**Published:** 2023-12-20

**Authors:** J. Mallet, P.-A. Billiet, M. Scarton, N. Benichou, M. Bobot, K. Chaibi, A. Hertig, J. Hadchouel, D. Dreyfuss, S. Gaudry, S. Placier

**Affiliations:** 1 French National Institute of Health and Medical Research (INSERM), UMR_S1155, CORAKID, Hôpital Tenon, Sorbonne Université, 75020 Paris, France; 2 Present Address: Intensive Care Unit, Service de Réanimation Médico-Chirurgicale, AP-HP, Hôpital Avicenne, 125 Rue de Stalingrad, 93000 Bobigny, France; 3 Service de Médecine Intensive Réanimation, Hôpital Henri Mondor, Assistance Publique Hôpitaux de Paris, Créteil, France; 4 Service de Médecine Intensive Réanimation, Hôpital Louis Mourier, Assistance Publique, Colombes, France; 5 Service de Néphrologie, Hôpital Européen Georges Pompidou, Assistance Publique Hôpitaux de Paris, Paris, France; 6 Université de Paris, Paris, France; 7 Centre de Néphrologie et Transplantation Rénale, Hôpital de la Conception, Assistance Publique Hôpitaux de Marseille, Marseille, France; 8 Aix Marseille Univ, INSERM 1632, INRAE 1260, C2VN, CERIMED, Marseille, France; 9 Health Care Simulation Center, UFR SMBH, Université Sorbonne Paris Nord, Bobigny, France

**Keywords:** Acute kidney injury, Intensive care unit, Renal replacement therapy, Hemodialysis, Adenine 0.75%, Animal model

## Abstract

**Background:**

Acute kidney injury (AKI) is frequent among critically ill patients. Renal replacement therapy (RRT) is often required to deal with severe complications of AKI. This technique is however associated with side effects such as hemodynamic instability and delayed renal recovery. In this study, we aimed to describe a novel model of hemodialysis in rats with AKI and depict a dialysis membrane performance.

**Methods:**

Eighteen Sprague–Dawley rats received 0.75% adenine-rich diet to induce AKI. After 2 weeks, nine underwent an arterio-venous extracorporeal circulation (ECC) (ECC group) for 2 h without a dialysis membrane on the circuit and nine received a hemodialysis session (HD group) for 2 h with an ECC circuit. All rats were hemodynamically monitored, and glomerular filtration rate (GFR) was measured by transcutaneous fluorescence after the injection of FITC-Sinistrin. Blood samples were collected at different time points to assess serum creatinine and serum urea concentrations and to determine the Kt/V. Sinistrin concentration was also quantified in both plasma and dialysis effluent.

**Results:**

After 2 weeks of adenine-rich diet, rats exhibited a decrease in GFR. Both serum urea and serum creatinine concentrations increased in the ECC group but remained stable in the HD group. We found no significant difference in serum creatinine and serum urea concentrations between groups. At the end of experiments, mean serum urea was 36.7 mmol/l (95%CI 19.7–46.9 mmol/l) and 23.6 mmol/l (95%CI 15.2–33.5 mmol/l) in the ECC and HD groups, respectively (*p* = 0.15), and mean serum creatinine concentration was 158.0 µmol/l (95%CI 108.1–191.9 µmol/l) and 114.0 µmol/l (95%CI 90.2–140.9 µmol/l) in the ECC and HD groups, respectively (*p* = 0.11). The Kt/V of the model was estimated at 0.23. Sinistrin quantity in the ultrafiltrate raised steadily during the dialysis session. After 2 h, the median quantity was 149.2 µg (95% CI 99.7–250.3 µg).

**Conclusions:**

This hemodialysis model is an acceptable compromise between the requirement of hemodynamic tolerance which implies reducing extracorporeal blood volume (using a small dialyzer) and the demonstration that diffusion of molecules through the membrane is achieved.

**Supplementary Information:**

The online version contains supplementary material available at 10.1186/s40635-023-00583-7.

## Background

Acute kidney injury (AKI), manifesting as an abrupt decline in renal function, is frequent among intensive care unit (ICU) patients [1]. The management of AKI is notably based on renal replacement therapy (RRT). This invasive technique may be associated with complications such as hemodynamic instability, complications relating to vascular accesses (i.e.,, infection or thrombosis), and impaired or delayed renal recovery [2, 3]. Indeed, recent studies have evidenced delayed renal function recovery in patients allocated to an early RRT initiation strategy [4, 5] or with a high intensity RRT [6, 7]. RRT could represent a “second hit” causing the onset of renal failure on previously damaged kidneys (by sepsis for instance) [3], leading to a maladaptive tubular repair and chronic kidney disease (CKD) [2]. This novel concept has been named *Artificial Kidney-Induced Kidney Injury* [3]. However, the precise determinants of RRT-induced kidney injury are poorly understood. Their elucidation requires an animal model of RRT. The first step of such program consists in setting an appropriate model of hemodialysis. Several animal studies tested models of RRT in rodents since the end of the seventies. Dialyzer membrane types included both cuprophan and more biocompatible material. The extracorporeal blood volume varied between from 1.5 to 5 ml and blood transfusion was frequently needed to ascertain hemodynamic stability. Details of previous models are provided in Additional file 1: Table S1.

The aim of the present study was to describe the model of hemodialysis developed in our laboratory and characterize dialysis membrane performance.

## Methods

### Animals

Sprague–Dawley rats aged 4–6 months old (JANVIER LABS, France) were used. Only male rats were used to avoid hormonal variations and allow sufficient weight of the animals, necessary for the implantation of vascular catheters and hemodynamic tolerance of extracorporeal circulation. They were acclimated for 1 week on ventilated racks in the animal facility of the laboratory. Rats had open access to water and 12-h night and day cycles.

Rats from a first group were placed on extracorporeal circulation (ECC) (ECC group) without dialysis membrane on the circuit for 2 h. Rats from a second group received hemodialysis (HD group) for 2 h with an ECC circuit. Both groups were evaluated after AKI induction.

This protocol was validated by the French Ministry of Research and Education (APAFIS number: 22263-2019100310367212v7).

### Induction of AKI

We chose an adenine-induced AKI model because of its relatively low variability compared to the other models of AKI classically used in rats. Moreover, adenine diet is a well-tolerated model of AKI in rats which avoids the stress, pain and blood loss of surgical model and thus helps to limit the mortality risk linked to the model.

Animals were daily fed with food enriched with 0.75% (percentage of animal weight) adenine. Adenine is a purine-like substance which causes acute renal failure by tubulo-interstitial crystalline nephritis when given at high dose in rodents [8, 9]. The rats had open access to water and 12-h night and day cycles. Duration of exposure was varied to determine the optimal duration of adenine feeding in preliminary experiments in 4 rats. Sequential glomerular filtration rate (GFR) measurements (Day 0, 7, 14, 21 of the 0.75% adenine rich diet) were obtained. Glomerular filtration rate was measured, and serum creatinine and serum urea were determined on the first day of the adenine feeding. The 2 groups of rats (ECC group and HD group) were studied after 2 weeks of adenine regimen. This regimen resulted in a dramatic decrease of GFR which dropped from 0.840 ± 0.145 ml/min/100 g to 0.118 ± 0.088 ml/min/100 g.

### Extracorporeal circulation and hemodialysis models

Forty-five minutes before surgical procedures, rats received analgesia with a subcutaneous injection of Buprenorphine at a dose of 0.1 mg/kg (VETERGESIC^®^ multidose 0.3 mg/ml solution injectable, CEVA Santé animale, France). They were then anesthetized by isoflurane inhalation (Vetflurane^®^, Virbac, France) induced at 3% in a chamber and maintained between 1.5 and 2.5% with a mask (Tem Sega, France) using an anesthetic gas vaporizer (MSS Industries). Areas to be catheterized were shaved beforehand and disinfected with 10% Polyvidone iodine (Vétédine 10%, Vetoquinol, France). Tissue dissection was done using small Moria scissors and forceps (FST, Germany) and vascular incisions were made with microsurgical scissors under optic microscopy in 10× magnification. Vascular accesses for ECC were obtained by inserting 22-Gauge catheters (BD InsyteTM Autoguard BC, USA) in the left carotid artery and the right femoral vein. Catheters were secured using a silk coil thread size 5.0 (FST, Germany). Cannulas were fixed to the skin using 4.0 sutures mounted on 16-mm needles (MersilkTM 4.0 FS-3, Ethicon, USA). Each catheterized area was finally covered with a previously warmed wet compress. All surgical procedures were performed by an engineer specialized in animal experimentation (SP).

**Fig. 1:**
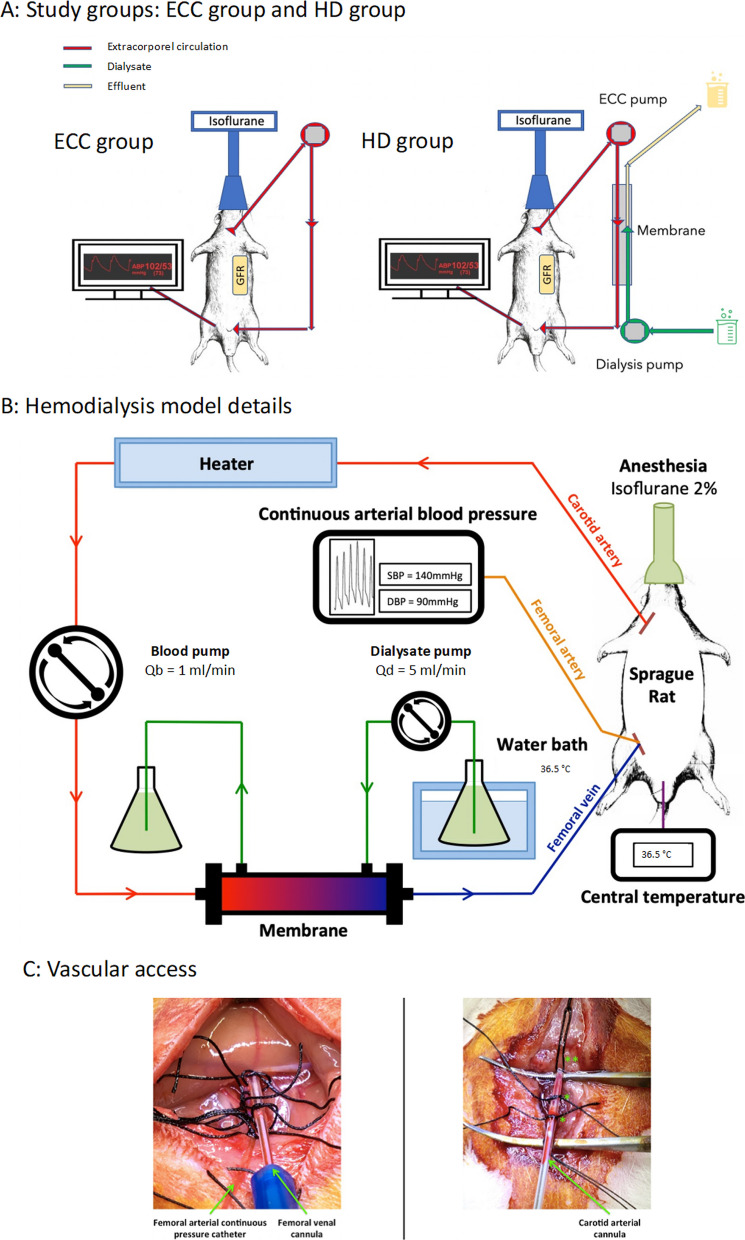
Experimental model. ECC: extracorporeal circulation, HD: Hemodialysis, GFR: glomerular filtration rate, Qs: blood flow; Qd: dialysate flow

Rats kept under anesthesia were then connected to an ECC circuit made of silicone tubing (Internal diameter 1 mm, Wall diameter 1 mm, CR Pumps, China) that connected carotid artery to femoral vein and driven by a peristaltic pump (BT100M/DG-1 [7], CR Pumps, China) previously calibrated at a flow rate of 1 ml/min. We initiated isovolemic ECC by connecting animals to a fully filled circuit with 1.5 ml normal saline 0.9%. In both groups, rats received a 2-h ECC session. In the HD group, a hemodialyzer was inserted in the circuit. Dialysis fluid, driven by a second pump (BT100M / DG-1 [7], CR Pumps, China) previously calibrated at 5 ml/min, flowed counter-currently to blood. The dialysis fluid composition was [Ca2+] 1.25 mmol/l, [Mg2+] 0.6 mmol/l, [Na+] 140 mmol/l, [Cl−] 115.9 mmol/l, [HPO42−] 1.2 mmol/l, [HCO3−] 30 mmol/l, and [K +] 4 mmol/l. The 2-h hemodialysis session was carried out without ultrafiltration using a 20-cm^2^ modified polyethersulfone membrane with 50-kDa pores (MidiKros^®^, Spectrum Laboratories, USA). Characteristics of the model are summarized in Fig. 1.

The entire circuit was pre-impregnated with heparin sodium (Panpharma^®^ 5000 UI/ml solution injectable IV, France) 100 IU/ml of saline solution 0.9% before being rinsed. A bolus of 0.2 ml of heparinized saline solution 0.9% was administered at connection. At the end of the session, blood in the circuit was restituted. The catheterized arterial and venous vessels were ligated using 5.0 silk threads previously placed around the vessels. Muscular and skin layers of the two surgical approaches were sutured by stitches made with Ethicon 4.0 thread. Postoperative analgesia was provided by a new subcutaneous injection of buprenorphine at a dose of 0.05 mg/kg approximately 1 h after the animal woke up.

### Monitoring

All rats underwent hemodynamic monitoring via a right femoral arterial catheter inserted as previously described (Polyethylene Tubing 0.011 × 0.024 × 0.0065, Phymep, France) containing heparinized 0.9% NaCl and connected to a pressure sensor (Blood Pressure Transducer, EMKA Technologies, France) at the beginning of the intervention. Systolic (SBP), diastolic (DBP) and mean (MBP) arterial blood pressure as well as heart rate (HR) values were collected using the IOX software (EMKA Technologies, France). Rats received crystalloid fluid expansion (0.9% NaCl) during procedure. It consisted of three intravenous standardized boli: 0.3 ml of purge at connection of the right femoral venous ECC catheter, 0.2 ml of heparinized NaCl 0.9% at priming of ECC, and 0.3 ml at 30 min of ECC. To this was added 1 or 1.5 ml filling with 0.9% NaCl at restitution depending on the length of the circuit (with or without dialysis membrane).

The target for MBP during procedures was set at a minimum of 65 mmHg. Additional intravenous NaCl 0.9% and/or intravenous norepinephrine were given to correct any hypotension.

Central temperature was monitored by a rectal probe (Temperature Controller, CMA/Microdialysis, Sweden). Rats were placed on a heating plate and exposed to a heating light throughout procedure to maintain normal core temperature. A heater was also installed on the arterial branch of the ECC, and the dialysate was warmed in a water bath at 36.5 °C before contact with the membrane. The expansion fluids were also warmed before administration. The body temperature objective was between 36 °C and 37 °C for all rats, to ensure satisfactory per-procedural hemodynamic stability. Finally, the rat recovery phase was carried out in an incubator at 28 °C.

### GFR measurement

Glomerular filtration rate was determined using transcutaneous monitoring of the decrease in fluorescence after intravascular injection of FITC-Sinistrin (batch VE17202021, Fresenius Kabi, Linz, Austria), an exogenous molecule of 5 kDa freely filtered by the kidney and not reabsorbed, coupled with FITC (Fluorescein 5(6)-isothiocyanate) [9]. The fluorescence detector, a miniaturized photodiode (MediBeacon GmbH, Mannheim, Germany), was attached to the left flank of the animal previously depilated and protected from any light source by placing occulting paper. A dose of 4 mg/100 g of body weight (concentration of 40 mg/ml) of Fluorescein-Isothiocyanate (FITC)-Sinistrin was injected via femoral artery 10 min after placing the camera to obtain a baseline value. The fluorescence peak was then obtained approximately 15 min after injection of FITC-Sinistrin. The camera measured the transcutaneous fluorescence emitted from the dermal capillaries over a total period of 3 h. The GFR was calculated by the MB Lab/MB Studio software (MediBeacon GmbH, Mannheim, Germany) from the slope of the decrease in fluorescence intensity over time according to a tri-compartmental model with linear regression [10, 11].

### Evaluation of hemodialysis efficiency

#### Kt/V measurement

Urea purification during ECC sessions is expressed by the measurement of Kt/V. Blood samples were collected at the time of ECC initiation (T0) and after 30 min, 60 min, and 120 min of ECC (respectively T30, T60 and T120). They were centrifugated at 4000 rpm for 10 min. Plasma was stored at − 80 °C. Serum creatinine and serum urea concentrations were secondly determined in the biochemical ward of Tenon Hospital (Paris). The Kt/V was estimated with the Daugirdas equation that includes pre- and post-dialysis serum urea, duration of dialysis, ultrafiltration volume, and post-dialysis weight [12].

#### Sinistrin elimination

In both groups, urine samples were collected from all rats at the end of ECC/dialysis and bladder volume was measured by collecting urine with a needle in two rats. In the HD group, effluent fluid was collected at the first minute (T1) and after 10, 20, 30, 60, and 120 min of dialysis (respectively T10, T20, T30, T60, and T120). Sinistrin concentrations were quantified by fluorescence measurement (*Tecan, Männedorf, Switzerland*) at a wavelength of 488 nm with a wavelength reference of 530 nm. The Sinistrin quantity was assessed by multiplication of the concentration with the total sample volume (e.g., effluent volume of 150 ml at 30 min with a 5 ml/min calibrated pump).

### Statistics

We did not perform a power calculation to determine the number of rats in each group. Quantitative variables are shown by means ± standard error of the mean (S.E.M.). Normality of distribution was assessed, and comparison tests were adapted. Statistical analysis was performed using Prism GraphPad Software (San Diego, California) and R (R Foundation for Statistical Computing, Vienna, Austria). All tests were two-sided.

To compare creatinine and serum urea levels, we used a two-way Analysis of Variance (ANOVA) with repeated measures. *p* values below 0.05 were considered statistically significant.

## Results

### Animals

Nine animals in each group were studied. There were 3 deaths during the procedure (2 in the ECC group and 1 in the HD group). Twelve rats (6 in the ECC group and 6 in the HD group) required norepinephrine infusion in addition to fluid administration to reach a 65 mmHg MBP goal.

### Adenine-induced kidney injury

**Fig. 2:**
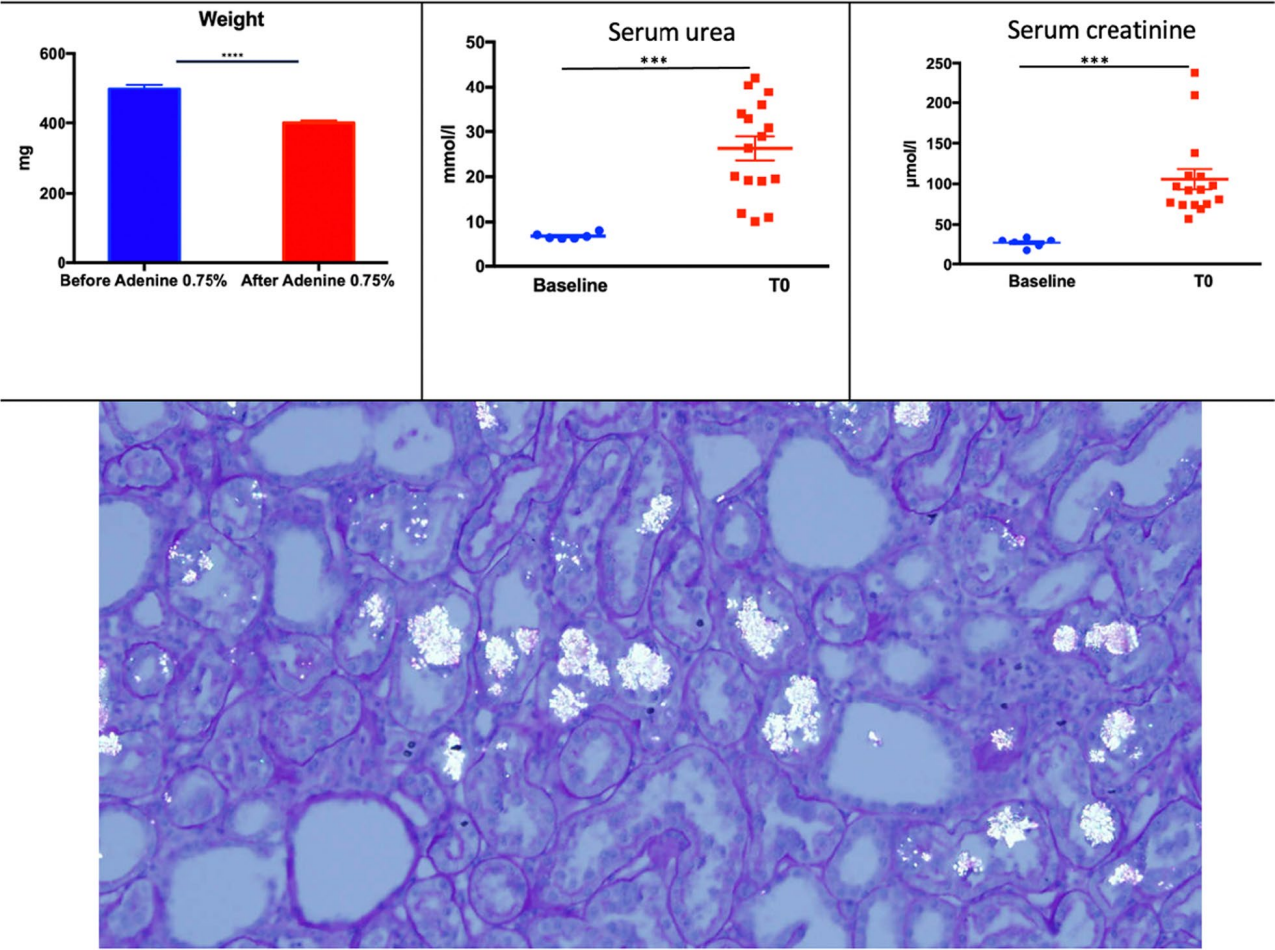
Adenine 0.75%-induced kidney injury after 2 weeks. Top-left panel shows the bodyweight loss, top-center and top-right panels respectively show blood urea nitrogen and serum creatinine concentration before and after adenine-rich diet. Bottom panel shows a PAS staining of a kidney section. Acute tubular necrosis signs are present alongside crystal’s deposits. ***p < 0.0001

After 2 weeks of adenine-enriched food, rats had a mean body weight loss from 497 mg (95%CI 472–526) to 395 mg (95%CI 385–415) (*p* < *0.0001*). Acute kidney injury was revealed by an increase in mean serum urea level from 6.7 mmol/L (95%CI 6.2 to 7.6) to 27.6 mmol/l (95%CI 20.6–32.0) (*p* < 0.0001) and of mean serum creatinine concentration from 29.0 µmol/l (95%CI 20.6–33.4) to 92.0 µmol/l (95%CI 74.7–122.9) (*p* < *0.0001*). Histological sections obtained in 4 additional rats used for AKI model development showed a pattern of acute tubular necrosis with dilated tubular lumens, loss of brush borders, and flattened epithelium of proximal tubules. Tubular crystal deposits were also observed (Fig. 2).

### Glomerular filtration rate measurement

**Fig. 3:**
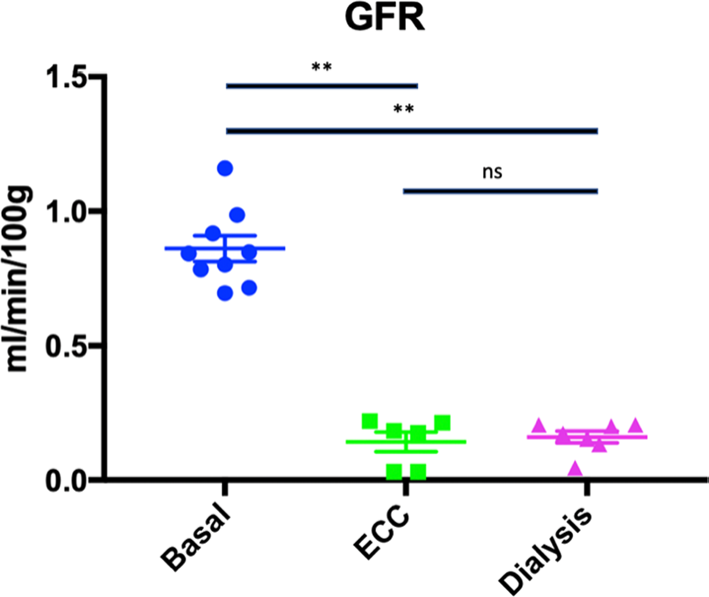
Glomerular filtration rate measurement by the transcutaneous FITC-Sinistrin method. GFR: glomerular filtration rate; ECC: extracorporeal circuit group; Dialysis: HD group ** *p* < 0.01

Prior to 0.75% adenine feeding, basal mean GFR assessed by the FITC-Sinistrin method was 0.842 ml/min/100 g (95%CI 0.749–0.972 ml/min/100 g). After 0.75% adenine diet, mean GFR decreased to 0.173 ml/min/100 g (95%CI 0.107–0.213 ml/min/100 g) and 0.179 ml/min/100 g (95%CI 0.05–0.234 ml/min/100 g) in ECC and HD groups respectively (*p* < 0.01) (Fig. 3).

### Dialysis efficiency

#### Biochemical parameters

**Fig. 4:**
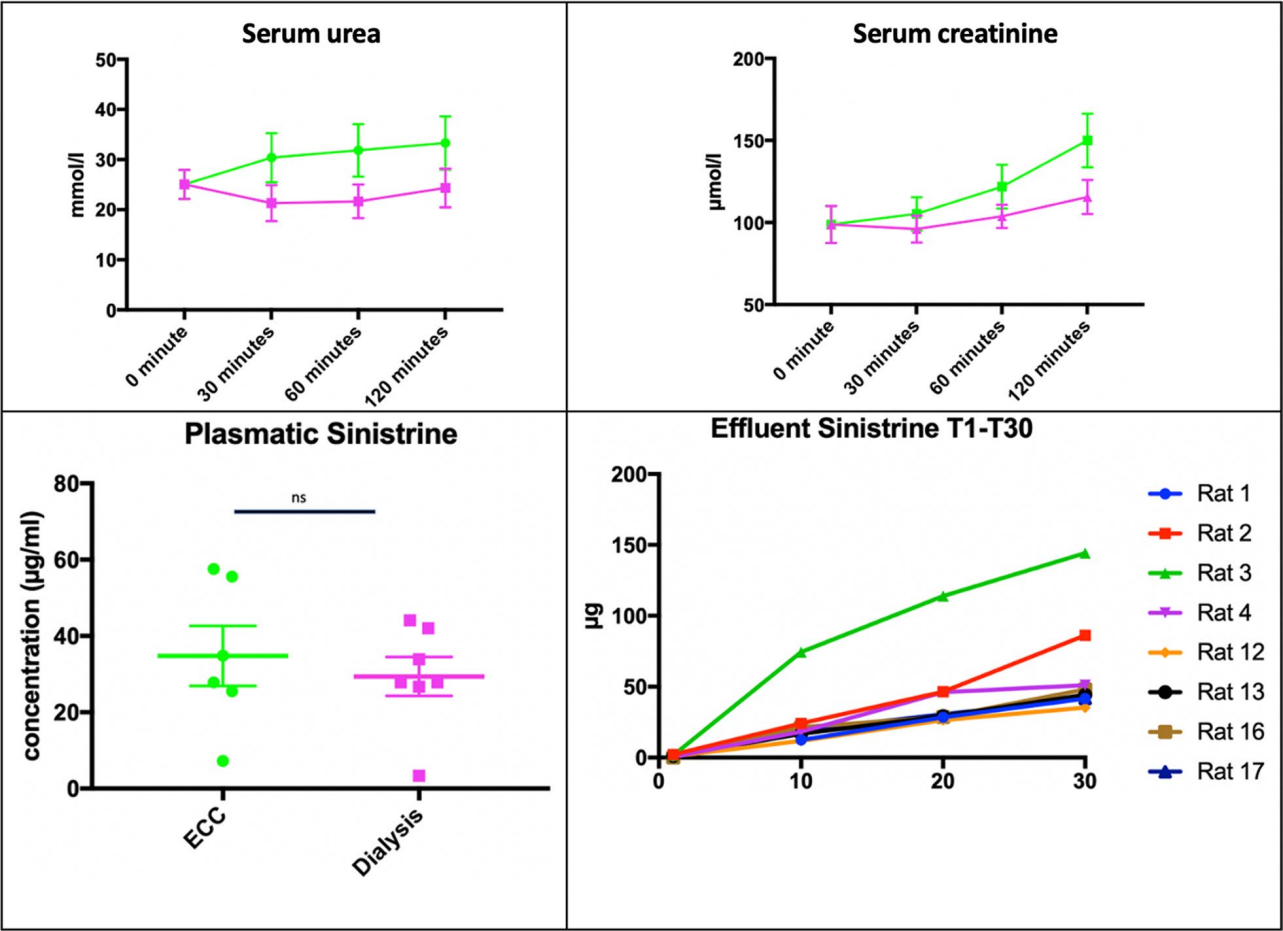
Membrane efficiency. ECC: extracorporeal circuit group; Dialysis: HD group. Top panels show serum urea and serum creatinine evolution during the procedure (ECC group: green curve; HD group: cyan group). Bottom left panel shows plasmatic concentration of Sinistrin at the end of the procedure. Bottom right panel shows the evolution of the Sinistrin quantity during the first 30 min of the dialysis

Both mean serum urea and serum creatinine concentrations increased in the ECC group but remained stable in the dialysis group during the experiments. Comparisons between groups using ANOVA test were not significant for serum creatinine (*p* = 0.09) and serum urea (*p* = 0.77). At the end of the ECC or dialysis session, mean serum urea was 36.7 mmol/l (95%CI 19.7–46.9 mmol/l) and 23.6 mmol/l (95%CI 15.2–33.5 mmol/l) in the ECC and HD groups, respectively (*p* = 0.15) and mean serum creatinine concentration was 158.0 µmol/l (95%CI 108.1–191.9 µmol/l) and 114.0 µmol/l (95%CI 90.2–140.9 µmol/l) in the ECC and HD groups, respectively (*p* = 0.11) (Fig. 4). The Kt/V was 0.23 using the Daugirdas equation, calculated using pre- and post-dialysis mean serum urea concentrations, with no ultrafiltration, and a post-dialysis weight of 400 mg.

#### Sinistrin

Sinistrin quantity in the ultrafiltrate raised steadily during the dialysis session. At T120, mean quantity was 149.2 µg (95% CI 99.69–250.3 µg). The mean serum Sinistrin concentration at the end of the procedure was 31.33 µg/ml in the ECC group and 27.81 µg/ml in the dialysis group. The difference did not reach significance (*p* = 0.65).

## Discussion

The present study describes a hemodialysis model in rats with AKI induced by an adenine-rich diet. Although Sinistrin ultrafiltrate concentration steadily raised during the dialysis session, we found no significant difference in serum urea and serum creatinine levels between the group of rats treated with hemodialysis and the group of rats placed under extracorporeal circulation without hemodialysis membrane. The Kt/V of the model was estimated at 0.23.

This is the second hemodialysis model to have been developed on animals suffering from AKI [13]. Previously published hemodialysis models focused on drug dialyzability [14] or on blood purification of pro-inflammatory substances, with the unproved idea of an improvement of the septic condition [15]. Unlike many other models, ours has the advantage of not requiring blood transfusion owing to the small volume of the dialyzer. Previously published models of hemodialysis in rats are summarized in Additional file 1: Table S1.

At first glance, the results of this study may seem disappointing regarding the absence of effective plasma purification of urea and creatinine. However, the gradual increase in Sinistrin concentration in the effluent during the session proves that the hemodialysis membrane used is capable of purifying small molecules (5 KDa). There are several possible explanations for the absence of any significant drop in serum urea and serum creatinine concentrations. First, we should acknowledge an insufficient surface area of the hemodialysis membrane we used. In fact, this 20 cm^2^ surface area is smaller than the membrane surface areas used in previously published models. The animal models summarized in Additional file 1: Table S1 used membrane surfaces ranging from 37 cm^2^ to 157 cm^2^. We used smaller volume dialyzers to obtain better hemodynamic stability and to avoid any blood transfusion that would interfere with the goals of our future study (interaction between animal blood and dialysis membranes). Second, insufficient mean serum urea and creatinine concentrations at RRT initiation (27.6 mmol/l and 92.0 µmol/l respectively) to create a sufficient gradient between blood and dialysis fluid may also explain these results. Third, another explanation could be the insufficient duration of RRT session and the lack of statistical power.

An intriguing result of this study is the rise of renal function markers (serum urea and creatinine) during the session (in both groups but more pronounced in the ECC group). To explore potential mechanisms, we measured the hematocrit before and after the procedure, but we found a hemodilution rather than a hemoconcentration (data not shown). In the hypothesis of a dosage interference with the Sinistrin, we measured urea and creatinine in the Sinistrin preparation and after mixing the preparation with plasma samples. These tests did not show an influence of the Sinistrin on the measurement of urea and creatinine. Alternative hypotheses to explain this intriguing result could be the general anesthesia and the animals’ position during the session. It has been described that inhaled isoflurane may induce a drop in renal blood flow, especially in already impaired kidneys [16]. This effect on the kidneys improved when the anesthetic agent was stopped. The supine position has also been shown to be associated with significant proteinuria and kidney dysfunction [17].

To summarize, we implemented the first model of hemodialysis in rats with adenine-rich diet-induced AKI. This model can be improved in several ways: using a larger dialysis membrane, initiating RRT sessions in rats with higher serum urea and serum creatinine levels and extending the duration of sessions. The fine tuning of an efficient hemodialysis model may help to explore the mechanisms underlying renal injury induced by RRT in ICU patients with AKI.

### Supplementary Information

**Additional file 1: Table S1.** Previously published models of hemodialysis in rats.

## Data Availability

All data generated or analyzed during this study are included in this published article.

## Abbreviations

AKI: Acute kidney injury
ATN: Acute tubular necrosis
CKD: Chronic kidney disease
DBP: Diastolic blood pressure
ECC: Extracorporeal circulation
FITC: Fluorescein 5(6)-isothiocyanate
GFR: Glomerular flow rate
HD: Hemodialysis
ICU: Intensive care unit
MBP: Mean blood pressure
RRT: Renal replacement therapy
SBP: Systolic blood pressure
SEM: Standard error of the mean
