# Supplementary material for: A model of hemodialysis after acute kidney injury in rats
Source: Intensive Care Med Exp. 2023 Dec 20;11:97. doi: 10.1186/s40635-023-00583-7 (PMC10733261; doi:10.1186/s40635-023-00583-7)
Supplement: Supplementary file 1 — Additional file 1: Table S1. Previously published models of hemodialysis in rats. [file 40635_2023_583_MOESM1_ESM.docx]

Table S1

| Study | RRT type | Membrane | Pump | ECC volume | Dialysate | Duration |
| --- | --- | --- | --- | --- | --- | --- |
| Röckel 1978 | Arteriovenous  dialysis | Cuprophan  35 cm^2^ | Blood 0.7-2 ml/mn  Dialysis 1-4 ml/mn | 1.5 ml |  | 3 hours |
| Silver 1992 and Galons 1996 | Arteriovenous  Dialysis | Cuprophane  hollow fiber  150 cm^2^ | Blood 1 ml/mn/100g, Dialysis 20 ml/mn | 2.5 ml | sodium 140  bicarbonate 25  potassium 4  chloride 124  calcium 1.25  magnesium 1.5  glucose 11 | 90 minutes |
| Kranzlin 1996 | Arteriovenous  Dialysis | Cuprophan, Hemophan, or polyacrylonitrile 50 cm^2^ | Blood 1 ml/mn  Dialysis 20 ml/h) | 1.25 ml | Bicarbonate buffered bath +/- urea | 60 minutes |
| Yorimitsu 2012 | Arteriovenous  Dialysis | Polysulfone,  hollow fibers (97 mm length)  46.7 cm^2^ | Blood 1 ml/mn,  Dialsysi 5 ml/mn |  | Sublood BS | 2 hours |
| Pittsburgh  (Peng, Rimmelé, Kellum 2012, 2013) | veino-venous Hemoadsorption | hemoads column 1 ml | Blood 0.8 à 1 ml/mn |  | 0 | 4 hours |
|  | Continuous  Hemofiltration | Cartouche hemofilter (5 ml) | Blood 1 ml/mn | 5 ml | 0 |  |
| Pittsburgh (Kellum 2004, Peng 2008) | arterio-venous  Hemoadsorpion | hemoads column 1 ml | No pump |  | 0 | 3 hours |
| Kida 2014 | Arteriovenous  dialysis | Polysulfone  145-cm2  pore size 50 kDa | Blood 4.25 ml/mn  Blood 14 ml/mn |  | acetate-free dialysate | 4 hours |
| Shinozaki 2015 | veno-veinous hemofiltration + artério-veinous ECC | AN69ST (Gambro) | Blood 3 ml/mn, filtration rate 20 or 60 ml/h |  | Reinjection : PrismaSATE BGK4/2,5 | 6 hours |
| Maeda 2017 | Arterioveinous hemofiltration | PEPA or CTA, 66 cm^2^  200 fibers (50 mm length) | Blood 1 ml/mn, UF 0.1 ml/mn, post dilution substution 0.1 ml/mn |  | Reinjection : Bicarbonate | 3 hours |
| Shi 2018 | Arteriovenous  dialysis | Polysulfone  250 fibers  157 cm^2^ | Blood 1 ml/mn  Blood 5 ml/mn)  UF = 0 |  | Bicarbonate | 4 hours |
